# Resistin Modulates Low-Density Lipoprotein Cholesterol Uptake in Human Placental Explants via PCSK9

**DOI:** 10.1007/s43032-022-00943-w

**Published:** 2022-04-25

**Authors:** Sonia Nava-Salazar, Arturo Flores-Pliego, Giovanni Pérez-Martínez, Sandra Parra-Hernández, America Vanoye-Carlo, Francisco Ibarguengoitia-Ochoa, Otilia Perichart-Perera, Enrique Reyes-Muñoz, Juan Mario Solis-Paredes, Salvador Espino y Sosa, Guadalupe Estrada-Gutierrez

**Affiliations:** 1 Department of Immunobiochemistry, Instituto Nacional de Perinatologia, Mexico City, Mexico; 2 Neuroscience Laboratory, Instituto Nacional de Pediatría, Mexico City, Mexico; 3 Department of Obstetrics, Instituto Nacional de Perinatologia, Mexico City, Mexico; 4 Department of Nutrition and Bioprogramming, Instituto Nacional de Perinatologia, Mexico City, Mexico; 5 Coordination of Gynecologic and Perinatal Endocrinology, Instituto Nacional de Perinatologia, Mexico City, Mexico; 6 Department of Human Genetics and Genomics, Instituto Nacional de Perinatologia, Mexico City, Mexico; 7 Clinical Research Branch, Instituto Nacional de Perinatologia, Mexico City, Mexico; 8 Research Direction, Instituto Nacional de Perinatologia, Mexico City, Mexico

**Keywords:** Resistin, LDLR, Pregnancy, Placenta

## Abstract

Maternal metabolic status influences pregnancy and, consequently, the perinatal outcome. Resistin is a pro-inflammatory adipokine predominantly expressed and secreted by mononuclear cells, adipose tissue, and placental trophoblastic cells during pregnancy. Recently, we reported an inverse association between maternal resistin levels and fetal low-density lipoprotein cholesterol (LDL-C). Then, in this work, we used a human placental explant model and the trophoblast cell line JEG-3 to evaluate whether resistin affects placental LDL-C uptake. Resistin exposure induced the transcription factor *SREBP-2*, *LDLR*, and *PCSK9* mRNA expression, and changes at the protein level were confirmed by immunohistochemistry and Western blot. However, for LDLR, the changes were not consistent between mRNA and protein levels. Using a labeled LDL-cholesterol (BODIPY FL LDL), uptake assay demonstrated that the LDL-C was significantly decreased in placental explants exposed to a high dose of resistin and a lesser extent in JEG-3 cells. In summary, resistin induces PCSK9 expression in placental explants and JEG-3 cells, which could be related to negative regulation of the LDLR by lysosomal degradation. These findings suggest that resistin may significantly regulate the LDL-C uptake and transport from the maternal circulation to the fetus, affecting its growth and lipid profile.

## Introduction

Resistin was initially described as an adipocyte-specific hormone with a role in insulin resistance in mice. In humans, resistin is mainly expressed in peripheral blood mononuclear cells and macrophages, and its expression in adipose tissue is located in inflammatory cells but not in adipocytes [1, 2]. Resistin plays a significant regulatory role in the systemic inflammatory response, and it has been associated with several pathologies such as obesity, diabetes, insulin resistance, hypertension, and dyslipidemias [3, 4].

The mechanisms of action and signaling pathways activated by resistin are currently being investigated. Melone et al. found that serum from obese individuals with high resistin levels (50 ng/mL) reduce low-density lipoprotein receptor (LDLR) in cultured hepatocytes, altering the optimal LDL-cholesterol (LDL-C) levels, which can lead to atherogenesis [5, 6]. Resistin increases the proprotein convertase subtilisin/kexin type 9 (PCSK9) [5, 6], and this protein interacts with the extracellular epidermal growth factor A (EGF-A) domain of the LDLR, preventing binding to LDL-C. The complex LDLR-PCSK9 is endocytosed and sent to lysosomal proteolysis, preventing the recycling of LDLR and diminishing LDL-C clearance [7, 8]. High PCSK9 protein levels and mutations in the EGF-A domain of LDLR have been associated with familial hypercholesterolemia and a high risk of cardiovascular disease; therefore, dysregulation of this protein is critical for dyslipidemias and associated diseases [9, 10].

Resistin modulates the expression of genes involved in cholesterol metabolism, such as the sterol-regulatory element-binding protein 2 (SREBP-2), a transcription factor considered a master regulator of cholesterogenesis and lipogenesis. Although studies in HepG2 cells and human, rat, and mouse hepatocyte models have demonstrated that resistin induces SREBP-2 expression [5, 11], the molecular mechanism involved in this effect has been less explored. Interestingly, in macrophages, resistin modulates the PPARγ-dependent PI3K/AKT signaling pathway [12], which is involved in activating SREBPs [13]. In addition, SREBP-2 regulates other genes as 3-hydroxy-3-methylglutaryl-coenzyme A (HMG- CoA) reductase, LDLR, and PCSK9, suggesting that resistin plays an essential role in the cholesterol metabolism.

Pregnancy is considered an inflammatory state due to the maternal physiological adaptations to provide adequate conditions for fetal growth, and the role of resistin is still controversial [14, 15]. During pregnancy, cholesterol has a relevant role in fetal development, since it is an essential component of cell membranes and a precursor for steroid hormones [16]. Cholesterol transport into the placenta from the maternal circulation is essential for a successful pregnancy [17], and low fetal blood cholesterol concentrations have been related to intrauterine growth restriction (IUGR) [18–20]. In turn, IUGR has been associated with elevated maternal serum and umbilical cord resistin levels [19, 21]. Individuals who were small or had restricted fetal growth at birth have elevated rates of coronary heart disease, high blood pressure, elevated cholesterol levels, and abnormal glucose and insulin metabolism later in life, which is known as the developmental origin of health and disease (DOHaD) hypothesis [22, 23]. Recently, we reported an inverse association between maternal resistin and cord-blood LDL-C, suggesting that the maternal metabolic status may play an active role in regulating the fetal lipid profile [24].

Maternal cholesterol must cross the apical side of the syncytiotrophoblast layer (STB) in the form of LDL- or HDL-cholesterol through the LDL receptor (LDLR) and the HDL receptor scavenger receptor class B type I (SR-BI), respectively [25]. Cholesterol is then released through cholesterol transporters such as ATP-binding cassette transporter A1 (ABCA1) and ABCG1 to the placental endothelial cells, reaching fetal circulation [26, 27]. The main proteins implicated in maternal cholesterol uptake and efflux to the fetal circulation have been identified in the human placenta, although most of the molecular mechanisms involved remain unclear.

Resistin modulates LDLR, and its role in maternal–fetal placental cholesterol transport has not been explored. Therefore, this study aims to evaluate whether resistin modulates LDL-C in the human placenta using an in vitro explant model and JEG-3 cells, as they have been reported to be a suitable model for the study of cholesterol supply from maternal lipoproteins to fetal tissues [28, 29]. The placental explants and cell cultures were treated with human recombinant resistin to examine the effect on LDLR, PCSK9 protein, and LDL-C uptake.

## Materials and Methods

This study was approved by the Institutional Review Board of the Instituto Nacional de Perinatologia in Mexico City (212250–3210-21002–05-16). Written informed consent was obtained from all enrolled women before the collection of samples.

### Specimens

Human placentas were obtained from 12 healthy term pregnancies undergoing scheduled cesarean section with no evidence of labor. Inclusion criteria included ≥ 18 years old, no comorbidities, normal pre-gestational body mass index (p-BMI: 18.5–24.9 kg/m^2^), without any treatment that affects carbohydrate or lipid metabolism, and fetus without structural congenital malformation.

### Human Placental Explants

Twelve placental biopsies, including maternal and fetal sides, were collected in cold, sterile, phosphate-buffered saline (PBS) solution and processed no more than 30 min after delivery. Under sterile conditions, the tissue was washed with PBS three times in order to remove blood, cut in small pieces (0.5 × 3.0 cm), and maintained in DMEM (Gibco, 11965–092) supplemented with 10% of heat-inactivated fetal bovine serum (FBS) (Gibco, 10437028), and 1 × amphotericin-B penicillin–streptomycin (Gibco, 15240) at 37 °C and 5% CO_2_. Five placentas were used for expression and immunofluorescence assays; 30 explants were obtained from each placenta to be exposed to 0, 10, 25, 50, and 100 ng/mL resistin in triplicate, as these concentrations cover a normal range of serum resistin levels. For immunoblot assays, four placentas were used; six explants were obtained from each placenta to be exposed at 0 and 50 ng/ml resistin in triplicate. Three placentas were used for the LDL-C uptake assay with the BODIPY-LDLR reagent, using six explants for two different resistin concentrations in triplicate.

### HepG2 Cell Culture

HepG2 cells is a cell line from hepatocellular carcinoma from a male patient of 15 years old of *Homo sapiens*. The cell line was propagated according to American Type Culture Collection (ATCC, Manassas, VA). Cells were maintained in Dulbecco’s minimum essential medium (Gibco, DMEM, 11965–092) supplemented with 10% of heat-inactivated FBS (Gibco, 10437028), and 1 × amphotericin-B penicillin–streptomycin (Gibco, 15240) at 37 °C and 5% CO_2_.

### JEG-3 Cell Culture

JEG-3 is a cell line from choriocarcinoma of *Homo sapiens*. The cell line was growth according to ATCC. Cells were maintained in DMEM (Gibco, 11965–092) supplemented with 10% of heat-inactivated FBS (Gibco, 10437028), 1 × amphotericin-B penicillin–streptomycin (Gibco, 15240), and 1 mM Sodium Pyruvate (Gibco, 11360) at 37 °C and 5% CO_2_.

### Cell Viability Assay

The XTT assay was used for cellular viability assessment (Thermo Fisher Scientific, Cell proliferation kit II, 11465015001). The assay is based on the cleavage of the yellow tetrazolium salt XTT to form an orange formazan dye by metabolically active cells and was realized according to the manufacturer’s instructions. After 24 h, the absorbance was determined by spectrophotometry (BioTek, Synergy HT) set at 550 nm. In the biopsy assay, the values were corrected by dry weight. In all cultures, cell viability was greater than 80% (data not shown).

### Resistin Treatment

Cells and placental explants were incubated with human recombinant resistin (Peprotech, 450–19) at different concentrations (0, 10, 25, 50, and 100 ng/mL) for 24 h. For immunoblot analysis and LDL-C uptake with BODIPY-LDLR, HepG2 and placental explant experiments were done with 50 ng/mL resistin, and for JEG-3 assays, 100 ng/mL resistin were used. For immunodetection, HepG2 and JEG-3 cells were grown in culture chambers. During resistin treatment, the FBS was replaced with 0.2% of lactalbumin hydrolysate. After incubation, the culture medium was removed, and cells and tissues were washed with PBS three times.

### Real-Time Quantitative PCR Analysis for LDLR, PCSK9, and SREBP2

After resistin treatment, total RNA was isolated from HepG2, JEG-3 cells, and placental explants using the RNeasy Mini Kit, and the RNeasy fibrous tissue mini kit (Qiagen, 74104, 74704), respectively. cDNA was synthesized using SuperScript III First-Strand Synthesis Super Mix (Invitrogen, 18080–400), according to the manufacturer’s instructions. Real-time RT-qPCR was performed using a StepOnePlus™ Real-Time PCR System. The TaqMan probes used were: *LDLR*, Hs00181192_m1; *PCSK9*, Hs03037355_m1; *SREBP2*, Hs01081784_m1, and eukaryotic *18S* *rRNA* (4319413E) as endogenous control. All measurements were performed in triplicate. Relative gene expression was calculated using the 2^−ΔΔCT^ method [30].

### LDLR and PCSK9 Immunofluorescence

JEG-3 and HepG2 cells were fixed in 4% paraformaldehyde (PFA 4%) for 25 min, after resistin treatments. Placental cultured explants were washed in PBS and fixed in PFA 4% at 4 °C overnight. After this time, placental explants were infiltrated with 15 to 30% sucrose, embedded in OCT compound, and frozen in liquid nitrogen to cut 10 μm thick sections in a cryomicrotome (Reichert Jung Cryocut 1800). Fixed cells and placental explants were permeabilized with 0.1% Triton-X in PBS for 5 min, 2 N HCl for 15 min, and washed three times in PBS. Nonspecific antibody reactivity was blocked with a solution of 3% bovine serum albumin, 10% goat serum, 0.3 M glycine, 0.1% Triton-X in PBS at 4 °C overnight. After removal of the blocking reagent, cells and placental explants were treated with anti-PCSK9 antibody (Abcam, ab84041) and anti-LDL Receptor antibody (Abcam, ab 52818) diluted 1:150 (v/v) in PBS and incubated 2 h at room temperature. Alexa Fluor 568-conjugated goat anti-mouse (Abcam, ab175473), and Alexa Fluor 488-conjugated goat anti-rabbit (Abcam, ab150077) secondary antibodies were used at dilution 1:500 and 1:1000 (v/v) in PBS, respectively, for 1 h at room temperature. Cells and placental tissues were rinsed with PBS, and nuclei was stained with 300 nM 4′,6-diamidino-2-phenylndole (BioLegend, DAPI, Dilactate, 422801) for 5 min; finally, they were rinsed with PBS and mounted with Anti-Fade fluorescence mounting medium (Abcam, ab104135). Immunofluorescence images were acquired by laser scanning in a confocal microscope LSM 510 Meta inverted, based on an Axiovert 200 M motorized microscope (Carl Zeiss, Oberkochen).

### LDLR and PCSK9 Immunoblot Analysis

Protein expression of LDLR and PCSK9 was assessed by Western blotting. Briefly, HepG2, JEG-3 cells, and placental explants were washed twice in ice-cold PBS, and resuspended in M-PER Mammalian Extraction Reagent (Thermo Scientific, 78505), and T-PER Tissue Protein Extraction Reagent (Thermo Scientific) supplemented with commercial protease inhibitor cOmplete ULTRA tablets EDTA-free (Roche, 04693159001) on ice for 15 min; the cell debris were removed by centrifugation. Protein measurements were performed using the Bicinchoninic Acid Kit (Pierce BCA Protein Assay Kit, 23225), according to the manufacturer’s instructions. Proteins (30 μg) were mixed with Laemmli Buffer (BIO-RAD, 161–0747) containing 0.1 M dithioerythritol (SIGMA, D-9680) and denatured by heating. Samples were loaded onto 10% SDS–polyacrylamide gel electrophoresis and run at 150 V for 5 h. Proteins were transferred onto polyvinylidene difluoride membranes (Thermo Scientific, PVDF Transfer Membrane, 88520) using a wet transfer system with buffer containing 20% methanol, 192 mM glycine, and 25 mM Tris at 90 V for 1.5 h. After transfer, membranes were blocked with 5% non-fat dry milk in PBS for 30 min and incubated overnight at 4 °C with primary antibodies against LDLR (1 μg/mL) (R&D Systems, MAB2148) and PCSK9 (0.5 μg/mL) (Invitrogen, PA5-79789) in PBS. Membranes were washed with PBS three times and incubated with horseradish peroxidase-conjugated secondary antibodies: goat anti-mouse IgG-HRP (Santa Cruz Biotechnology, sc-2005) and goat anti-rabbit IgG-HRP (Santa Cruz Biotechnology, sc-2004) in PBS-T (PBS, 0.1% Tween 20) for 1 h at room temperature. After washing with PBS-T, immunoblots were revealed by chemiluminescence (Immobilon Western Chemiluminescent HRP Substrate, WBKLS0500). Relative protein levels were calculated based on GAPDH (Santa Cruz biotechnology, INC, sc-137179) for HepG2 cells and human placental explant; ACTIN (Santa Cruz biotechnology, INC, sc-47778) was used for JEG-3 cells. Images were acquired using a ChemiDoc Imaging system and the densitometric analysis of the bands was performed using Image Lab software (version 6.0.1).

### LDL-C Uptake with BODIPY-LDLR

Placental explants and HepG2 cells were treated with 50 ng/mL resistin, and JEG-3 with 100 ng/mL for 21 h. Then, cells were pre-treated with Heparin at 250 μg/mL (Inhepar, 5000 UI/mL) for 30 min at 37 °C. Bodipy FL LDL (Thermo Scientific, L3483) was added to the medium (10 μg/mL), and the cells and placenta explant cultures were incubated for 3 h. Cells and placental explant were rinsed with PBS, and nuclei was stained with DAPI (BioLegend, 422801) for 5 min. Cells and placental explants were fixed, treated, and mounted according to the immunodetection protocol. Relative fluorescence intensity was quantified using ImageJ software (Version 1.5Oi, National Institutes of Health, Bethesda, Maryland) and was normalized with the nuclei (DAPI).

### Statistical Analysis

Each experiment was performed with at least three different placentas using triplicates. The data were expressed as the mean ± standard deviation (SD) of at least three independent experiments. Differences between groups were evaluated using unpaired Student’s *t*-test or one-way analysis of variance (ANOVA), using the GraphPad Prism software (version 6.0c). A statistically significant difference was considered with a *p* < 0.05.

## Results

Human placentas were obtained from twelve healthy women. Seven newborns were male, and five were female. Demographic and clinical characteristics of donors were age 29.55 ± 5.85 years, *p*-BMI 21.85 ± 1.52 kg/m^2^, gestational weight gain 12.16 ± 4.21 kg, gestational age at sampling 38.8 ± 1.8 weeks, and fasting glucose 82.3 ± 19.5 mg/dL.

### Resistin Treatment Induces SREBP-2, LDLR, and PCSK9 Expression in Cell Cultures and Placental Explants

**Fig. 1:**
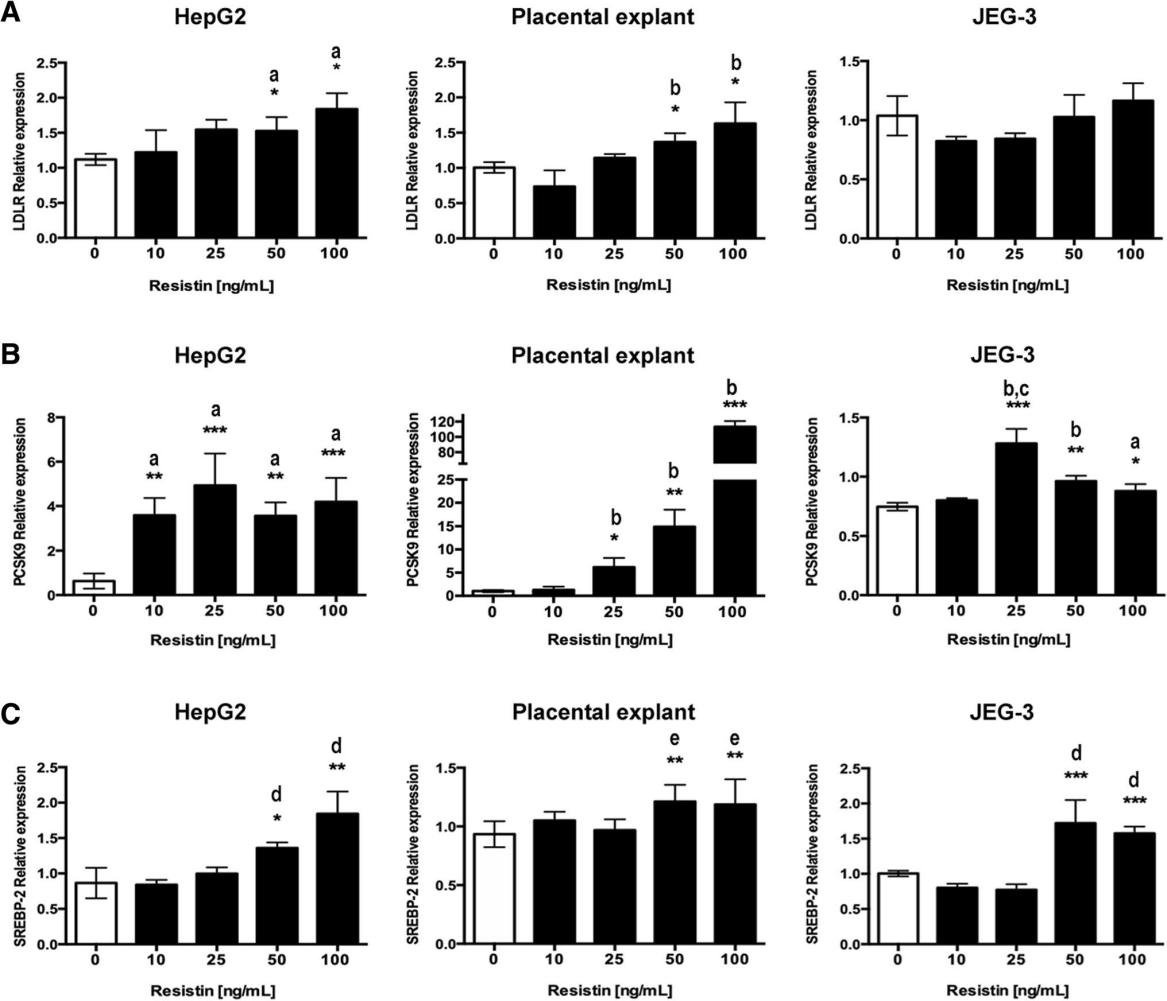
Resistin induces LDLR, PCSK9, and SREBP-2 expression. **A** *LDLR* expression significantly increased in HepG2 cells and placental explant. **B** *PCSK9* and **C** *SREBP-2* expression increased significantly in the three experimental models. A one-way ANOVA and Tukey post hoc test was performed for significance testing. Data are shown as mean ± SD of 5 independent experiments. a: vs. 0 ng/mL; b: vs. 0 and 10 ng/mL; c: vs. 50 and 100 ng/mL; d: vs. 0, 10 and 25 ng/mL, and e: vs. 0 and 25 ng/mL. *p < 0.05; **p < 0.01; ***p < 0.001

The expression of *LDLR*, *PCSK9*, and *SREBP-2* was evaluated by RT-qPCR in human placental explants and JEG-3 cells after exposure for 24 h to growing doses of resistin (0, 10, 25, 50, and 100 ng/mL), using HepG-2 cells as a positive control [5] (Fig. 1). A significant dose–response induction of *LDLR* expression was found in HepG2 cells and placental explant, at the highest resistin concentrations (50 and 100 ng/mL) vs. control (*p* ˂ 0.05). In JEG-3 cells, no significant increase in *LDLR* expression was observed (Fig. 1A). *PCSK9* expression was not dose-dependent in the cell lines, and the highest expression was observed at 25 ng/mL of resistin (*p* ˂ 0.001) (Fig. 1B). In contrast, a dose-dependent *PCSK9* expression was found in the placental explant exposed to resistin, and at 100 ng/mL of resistin, *PCSK9* expression increased more than a 100-fold vs. control (*p* ˂ 0.001). *SREBP-2* expression increased significantly at 50 and 100 ng/mL, regardless of the cell model (Fig. 1C).

### Resistin Decreases LDLR and Increases PCSK9 Protein Expression

**Fig. 2:**
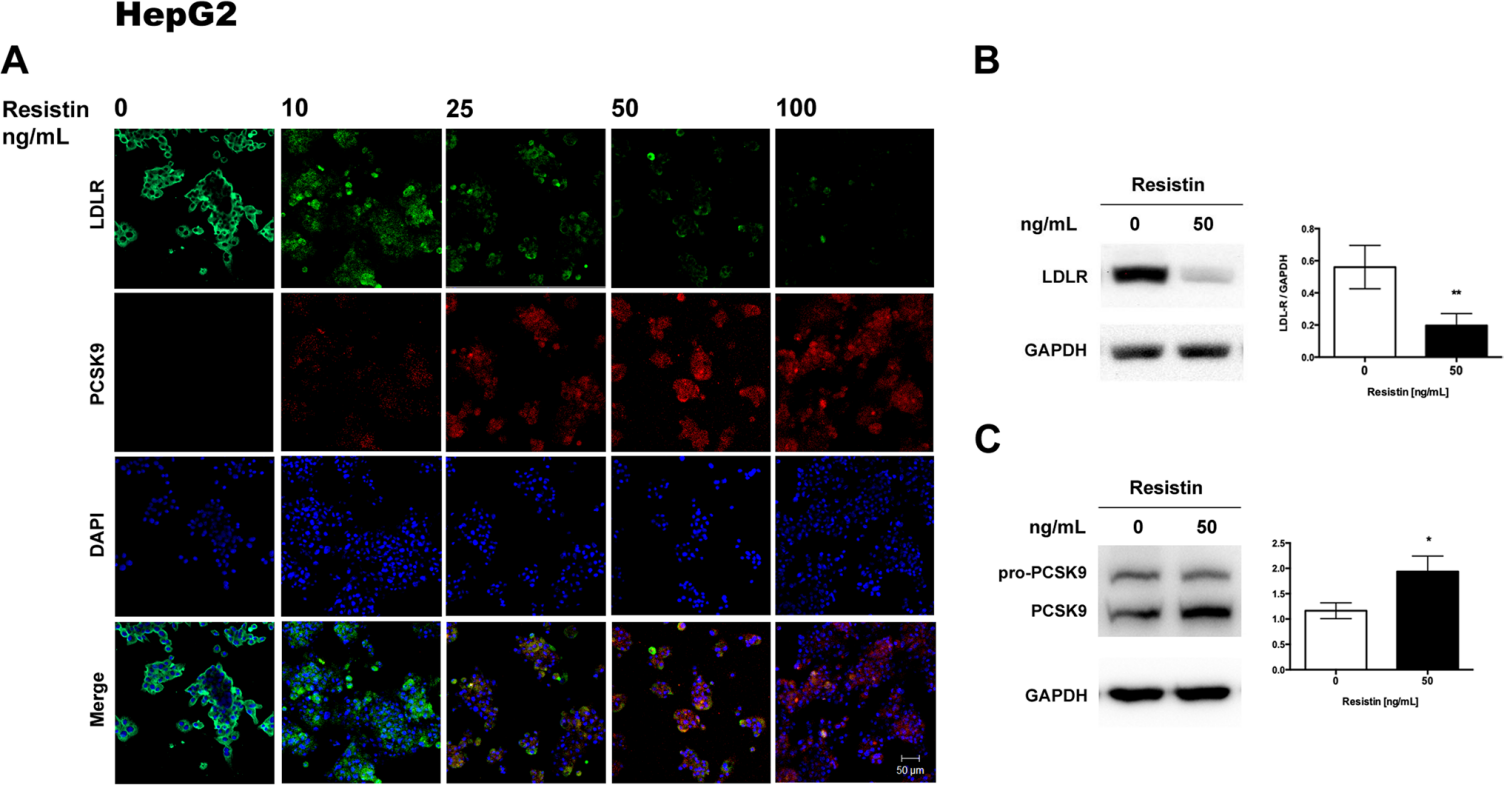
Resistin treatment in HepG2 cells downregulates LDLR and increases PCSK9 protein*.* **A** Representative immunofluorescence images showing that resistin reduced LDLR protein levels and increased PCSK9 in a dose-responsive manner (*n* = 5). **B** Representative Western blots of LDLR and **C** PCSK9; resistin inversely modulates both protein levels and was statically significant (*n* = 4). Immunofluorescence images 20 × magnification, scanning zoom 2 × . Scale bar represents 50 µm. Student’s *t*-test was performed for significance testing. Data are shown as mean ± SD. *p < 0.05; **p < 0.01

**Fig. 3:**
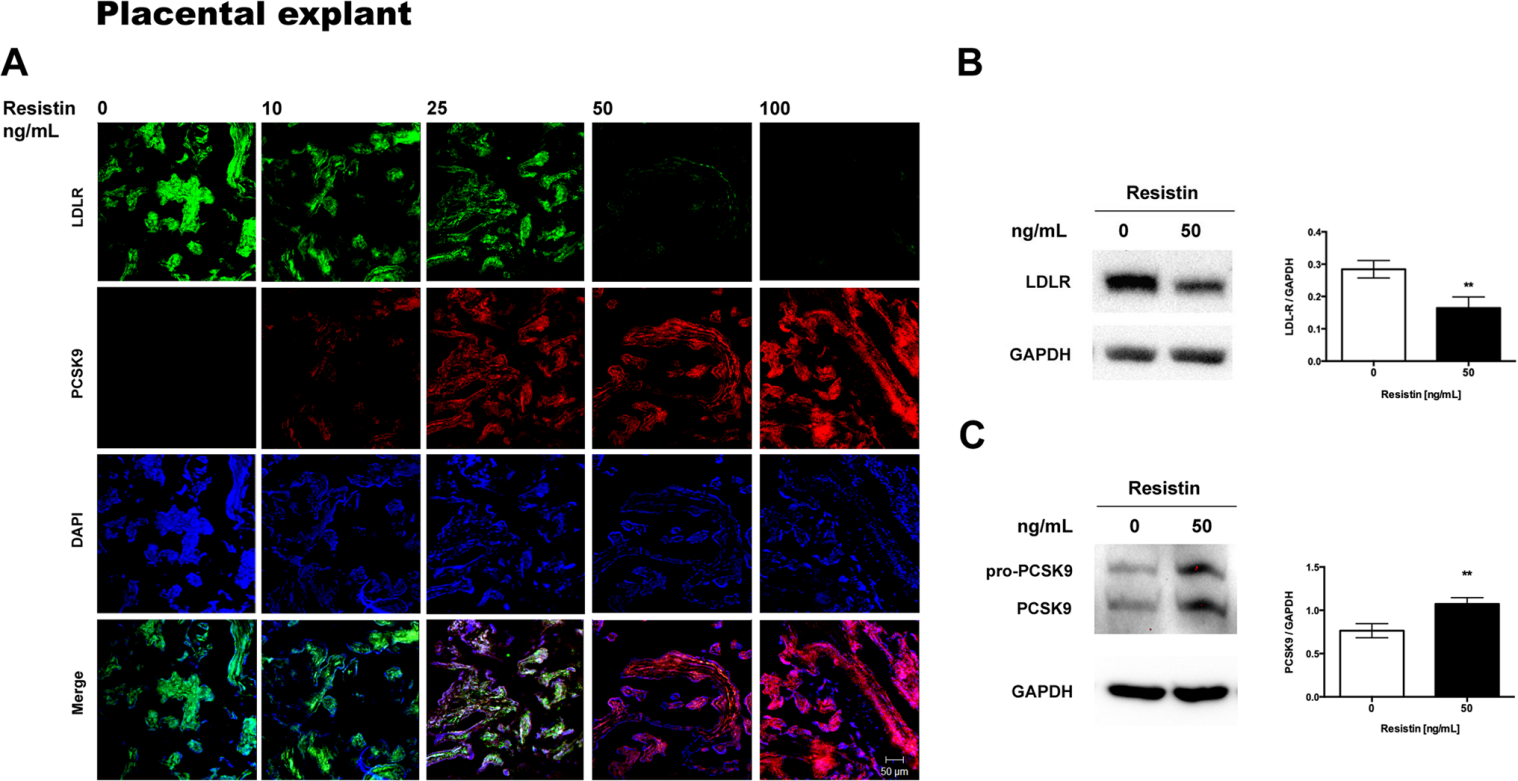
Resistin treatment in human placental explant culture cells downregulates LDLR and increases PCSK9 protein. **A** Representative immunofluorescence images (*n* = 5). **B** Representative Western blots of LDLR and **C** PCSK9 (*n* = 4). Immunofluorescence images 20 × magnification, scanning zoom 2 × . Scale bar represents 50 µm. Student’s *t*-test was performed for significance testing. Data are shown as mean ± SD. **p < 0.01

**Fig. 4:**
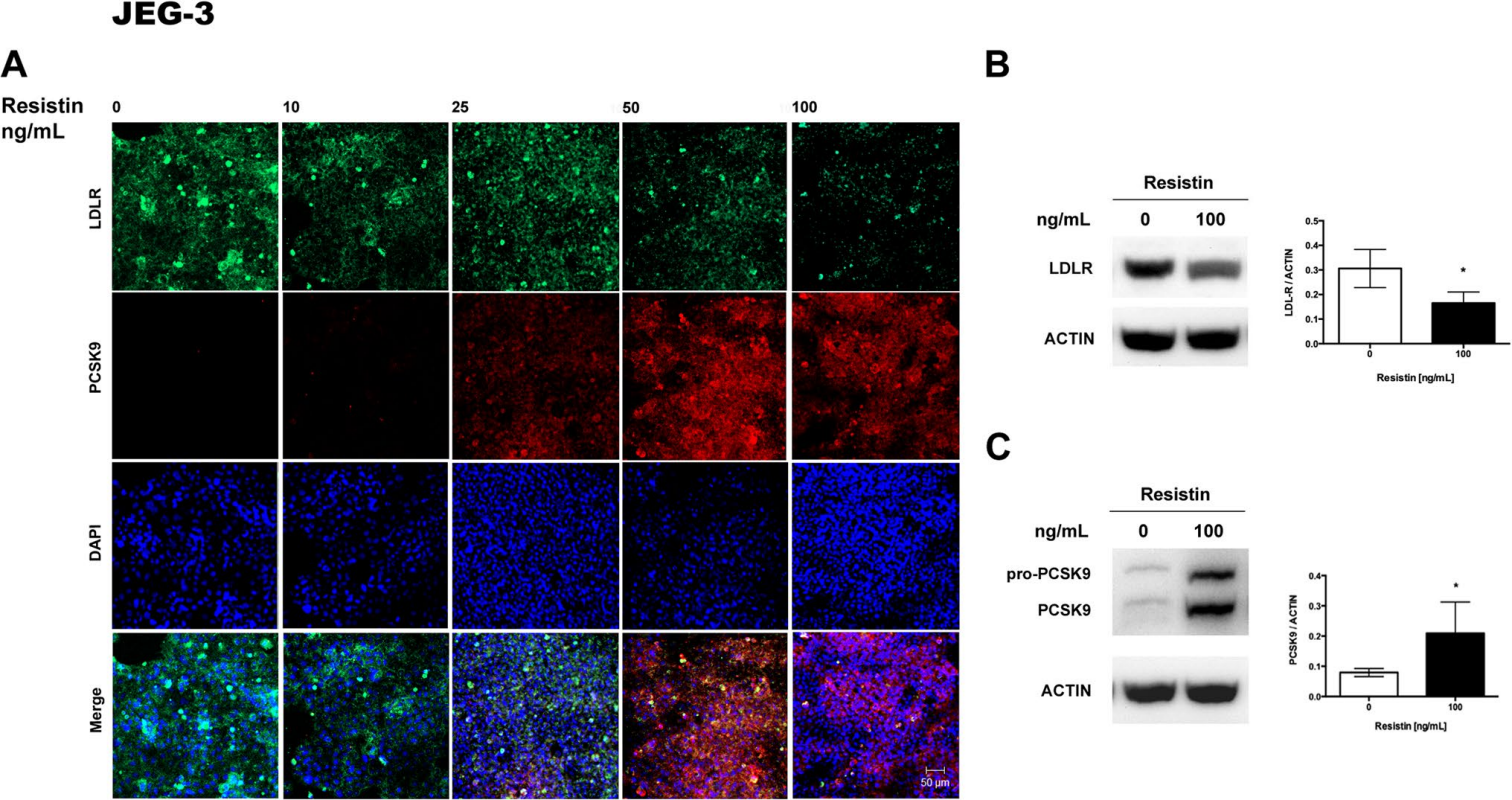
Resistin treatment in JEG-3 cells increases PCSK9 protein and partially decreases LDLR. **A** Representative immunofluorescence images show that a higher resistin concentration is necessary to reduce LDLR and increase PCSK9 protein levels (*n* = 5). **B** Representative Western blot of LDLR and **C.** PCSK9 protein (*n* = 4). Immunofluorescence images 20 × magnification, scanning zoom 2 × . Scale bar represent 50 µm. Data are shown as mean ± SD. *p < 0.05

PCSK9 and LDLR were localized by immunofluorescence in placental explants and cultures of HepG2 and JEG-3 cells after treatment with resistin. HepG2, human placental explant, and JEG-3 cells expressed LDLR in baseline conditions, mainly in the cytoplasm, as shown by immunofluorescence (Figs. 2A, 3A, and 4A). The expression of LDLR decreases when exposed to 50 and 100 ng/ml of resistin in placental explants and cell lines. Contrary, resistin induces the expression of PCSK9 protein, which was observable from 25 ng/mL and appears to be dependent on resistin concentration (Figs. 2A, 3A, and 4A).

In order to semi quantify the changes in LDLR and PCSK9 expression, Western blots were performed for each protein after incubation with resistin. For HepG2 cells and human placental explant cultures, exposure to 50 ng/ml of resistin was chosen due to the marked decrease of LDLR protein observed by immunofluorescence. For JEG-3, the highest concentration of resistin was used since a lower effect was observed on LDLR expression by immunohistochemistry when compared with HepG2 cells and placental explant. Western blots confirmed that resistin significantly decreased LDLR expression in placental explant and in both cell lines (Figs. 2B, 3B, 4B).

Human PCSK9 is synthesized as a precursor, pro-PCSK9 (75 kDa), that undergoes autocatalytic cleavage in the endoplasmic reticulum (ER). The mature form of PCSK9 (63 kDa) remains noncovalently bonded to its inhibitory prosegment and is released from the ER as a complex (62 + 13 kDa), which is secreted into the bloodstream [31]. Pro-PCSK9 and PCSK9 were identified by Western blot, but quantification was focused only on the active form of the enzyme, confirming that resistin significantly increased PCSK9 expression in placental explant and both cell lines (Figs. 2C, 3C, 4C).

### Resistin Treatment Decreased LDL-Cholesterol Uptake in HepG2, Human Placental Explant, and JEG-3 Cells

**Fig. 5:**
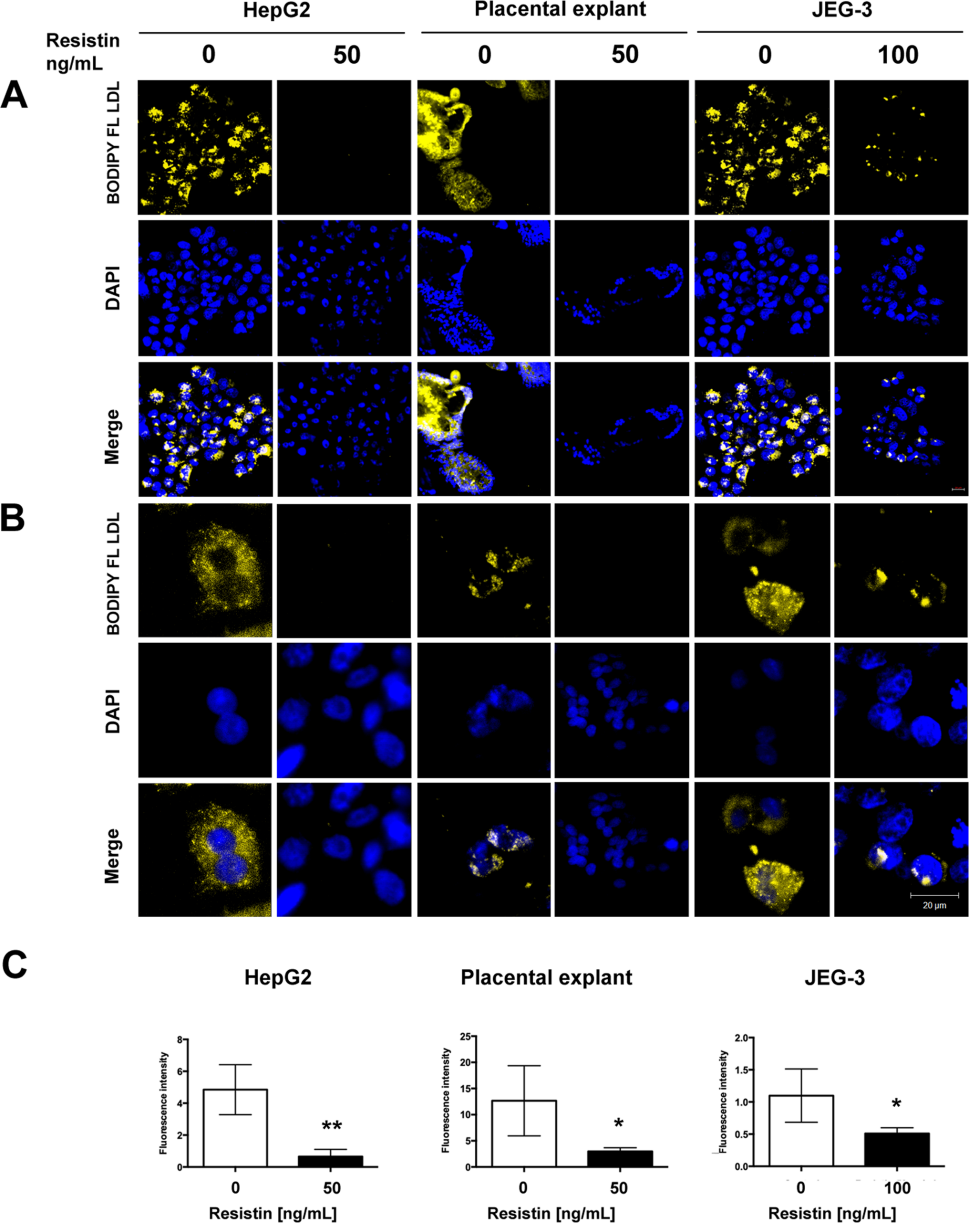
Resistin treatment decreases cholesterol LDL uptake in HepG2, human placental explant, and JEG-3 cells. **A** Representative immunofluorescence images of BODIPY FL LDL-cholesterol efflux assay, 20 × and **B** 40 × , Scale bar represents 20 µm. **C** Fluorescence intensity was quantified using ImageJ software and was normalized with the nuclei (DAPI). Student’s *t*-test was performed for significance testing. Data are shown as mean ± SD from three independent experiments. *p < 0.05; **p < 0.01

In order to evaluate the effect of lower LDLR expression on the LDL-cholesterol uptake, an assay with labeled LDL-cholesterol (BODIPY FL LDL) was performed. Using 50 ng/mL of resistin concentration in HepG2 and human placental explant, the labeled LDL-cholesterol within the cells significantly decreased (5 and 3 times, respectively). In JEG-3 cells, treatment with 100 ng/mL of resistin decreased labeled LDL-cholesterol by about 50% (Fig. 5).

## Discussion

Resistin is an adipokine that regulates glucose and lipid metabolism, tissue sensitivity to insulin, and systemic inflammation [32, 33]. Maternal resistin levels in plasma are higher than in non-pregnant women, and they have been positively correlated with gestational age [34–36] and maternal weight gain [37]. The placenta is a source of this hormone, which is mainly synthetized by the trophoblast cells [38, 39], and it has been suggested that placental resistin contributes to the physiological decrease in insulin sensitivity occurring in the second half of human pregnancy [40]. Several studies have associated high maternal resistin levels with more abdominal fat, and gestational diabetes [41], while low resistin levels have been associated with preeclampsia, but controversy exists between studies [36, 42–45].

The effects of maternal resistin levels in fetal development and maternal–fetal cholesterol transport have been poorly explored. Cholesterol is essential for embryo growth and development. In the fetus, cholesterol is derived from de novo synthesis, with a second source coming from the maternal circulation [46, 47]. The importance of the alterations in the cholesterol transport from mother to fetus and the changes in placental cholesterol metabolism that impact fetal development are not fully understood. Cholesterol is transported as lipoproteins, and the human placenta expresses LDLR, LRP-1 (LDL receptor–related protein 1), SR-BI, VLDL receptor (very low-density lipoprotein receptor), and the LRP8 (apoE receptor) [48, 49]. LDL particles contain 60–80% lipids circulating in human plasma, which are carried to the tissues [50]. Deficiencies in its transport may affect the developing fetus since the deregulation of placental steroid hormone synthesis could affect the maintenance of an adequate placental function [27, 51].

In this work, resistin increased the SREBP-2 expression in human placental explants and JEG-3 cultures. SREBP-2 is a basic-helix-loop-helix leucine zipper transcription factor that regulates genes involved in the synthesis and cellular uptake of cholesterol. SREBP-2 is considered a sensor of cholesterol levels, and in cholesterol-depleted cells, SREBPs are activated by two proteases to release a soluble fragment, then it goes to the nucleus to induce the transcription of its target genes such as LDLR and enzymes of cholesterol biosynthesis. Once LDL-derived cholesterol increases, SREBP activation by proteolysis is blocked and its target genes are downregulated [52]. In placental explants and HepG2 cells, *SREBP-2* and *LDLR* expression increased significantly at the 50 and 100 ng/mL of the resistin concentration. In JEG-3 cells, *SREBP-2* expression increases at the same doses as the other models. However, the *LDLR* expression, even with high-dose resistin treatment, did not change.

PCSK9, a negative regulator of LDLR, contains a sterol-regulatory element (SRE), and its expression can be regulated by SREBP-1 and SREBP-2; moreover, in vivo studies have suggested that PCSK9 is regulated predominantly by SREBP-2 [53]. Positive regulation of SREBP-2 by resistin can induce the expression of both LDLR and PCSK9. In the models used in this study, resistin treatment increases PCSK9 protein levels in a dose-dependent manner in placental explants and JEG-3 cells, decreasing the LDLR protein levels. As described, PCSK9 binds to LDLR, and the complex is endocyted and degraded, preventing the receptor from recycling. The LDLR is responsible for the binding and cellular uptake of LDL-C; the LDL-LDLR complex is internalized by endocytosis and dissociated by changes in pH. Free LDLR is recycled to the cell surface, while LDL-C is degraded in lysosomes to be used as a precursor for various cellular vital molecules [54, 55].

Melone et al. [5] also found that LDLR degradation does not depend exclusively on PCSK9, as they observed that resistin maintains the effect of reducing LDLR levels in PCSK9 knockout mice hepatocytes. Resistin also has a C-terminal cysteine-rich domain (CRD) that has homology to PCSK9, so it could compete with the same receptor, such as LDLR [56], and could induce LDLR degradation in a similar way to PCSK9, although so far, there are no conclusive studies.

Our results showed that in placental explants and JEG-3 cells, LDLR protein levels were reduced after exposure to resistin in a very similar way to the observed and described for HepG2 cells. However, for JEG-3, a higher resistin concentration (100 ng/mL) was needed to reduce the LDLR protein expression. JEG-3 cells highly express the SR-BI (scavenger receptor, class B type I), which plays a crucial role in cholesterol transport, mainly of HDL-derived cholesteryl esters, into cells and tissues. A study in murine ovarian granulosa cells found that the SR-BI can compensate for the loss of LDLR function. It is also known that in the first trimester of pregnancy, the expression of LDLR mRNA decreases compared to the third trimester, and SR-BI could be an alternative pathway for cholesterol supply during fetal development [29, 57, 58]. Placental explants in this study were obtained from term pregnancies, so our results suggest that at this point, cholesterol supply depends mainly on LDLR, and maternal resistin changes could be critical for fetal growth.

Considering the effect of resistin in the expression of the main proteins implicated in cholesterol transport, we evaluated the LDL uptake in human placental explant and JEG-3 using labeled LDL-cholesterol (BODIPY FL LDL). Resistin exposure significantly decreased LDL-cholesterol uptake in human placental explant when compared with control groups. According to the results in JEG-3 cells, a higher resistin concentration was necessary to reduce LDL-C uptake. The effect of exogenous resistin on LDL-C uptake in both systems suggests that maternal resistin affects the fetal environment.

We found that 50 ng/mL of resistin has a significant effect on the proteins involved in cholesterol uptake. Resistin levels in pregnant women has been reported in wide ranges (1.6 to 159 ng/mL) and are apparently influenced by some pathologies and even by ethnicity [34, 59]. Women in the first trimester of pregnancy have higher levels of resistin in plasma than non-pregnant women, with a significant increase at term. Because of its role in regulating insulin sensitivity, the relationship between maternal resistin levels and gestational diabetes has been evaluated in several studies, but the association is not conclusive due heterogeneity among them [60]; in the Chinese population [61], resistin values up to 62.38 ± 13.6 ng/mL have been found in women with this pathology. As high resistin levels in the first trimester of pregnancy (up to 36.55 ng/mL) have been associated with an increased risk of developing preeclampsia, it has been proposed that resistin concentration could be added to the predictive and prognostic algorithms for this pathology [62].

Our results suggest that resistin could be responsible for LDLR degradation by increasing PCSK9 protein expression, resulting in decreasing cholesterol uptake from the maternal circulation to the placenta and fetus. LDL contains apoprotein (20–22%), triglycerides (10–15%), phospholipids (20–28%), cholesteryl esters (37–48%), and cholesterol (8–10%), which are crucial for normal fetal development; in addition, the maternal nutritional status during gestation has been related to fetal growth [63]. Interestingly, LDL-C from the maternal bloodstream is the main precursor for the synthesis of placental progesterone [64], and low serum progesterone in the first trimester is a significant risk factor for low birth weight and possibly other placental dysfunction disorders such as hypertensive disorders of pregnancy [65].

All these findings are relevant because changes in maternal resistin levels could be one of the mechanisms implicated in intrauterine growth restriction (IUGR) or having a small for gestational age (SGA) newborn since several studies have reported a correlation between maternal resistin levels and these pathologies [66, 67]. Different studies have shown that serum resistin levels in the umbilical cord are higher in IUGR while lower in the macrosomic fetus. Also, an association between IUGR and decreased LDLR, low fetal LDL-C, and SR-BI has been reported [19]. These studies would suggest that resistin participates in the regulation of fetal growth. However, other studies have failed to find an association between maternal resistin and birth size, probably due to differences in the type of population included or the sample size [68, 69].

Although in this work we were only focused on the LDL-C uptake via LDLR regulation, cholesterol as a source of nutrients and precursor of hormones and cellular components can be taken from other lipoproteins using different transporters, such as those from the ABC, LRP family, and SR-B1 [27, 70] that were not studied in this work, so further studies are needed to evaluate the impact of elevated maternal resistin levels on other cholesterol transporters. Finally, some studies have related PCSK9 concentration to the gender of the newborn [71, 72], but in our work the number of placentas used in each experiment does not allow to perform a statistical analysis by gender, which represents a weakness of this study.

In conclusion, our work shows that resistin negatively regulates LDL-C uptake, which is one of the primary energy sources that the fetus obtains from the maternal circulation, to be used as a component of cellular membranes and precursor of molecules involved in multiple biological processes that are essential for development. The LDL-C transport impairment caused by increased resistin may affect energy balance and fetal growth.

## Data Availability

The data underlying this article are available in the article. Additional data underlying this article will be shared on reasonable request to the corresponding author.

## Declaration

### Research Resource Identifiers

Software:

GraphPad Prism, RRID:SCR_002798.

Image Lab Software, RRID:SCR_014210.

ImageJ, RRID: SCR_003070.
